# The Usage of the Term “Social” in Japanese Primary Care Literature

**DOI:** 10.7759/cureus.51972

**Published:** 2024-01-09

**Authors:** Junki Mizumoto, Masashi Izumiya, Shoko Horita, Masato Eto

**Affiliations:** 1 Department of Medical Education Studies, International Research Center for Medical Education, Graduate School of Medicine, University of Tokyo, Tokyo, JPN; 2 Department of Medical Education, School of Medicine, Teikyo University, Tokyo, JPN

**Keywords:** family medicine, terminology, qualitative research, primary healthcare, document analysis

## Abstract

Introduction: There has been a growing recognition of the importance of incorporating a "social" perspective in primary care practice. However, the specific meaning of the term "social" in the context of primary care is often not clearly defined or explained in the literature. This study aims to explore the usage and interpretation of the term "social" in primary care discourse in Japan.

Methods: We collected papers containing the term "social" ("shakai-teki" in Japanese) from 810 papers published between 2010 and 2022 in the Official Journal of the Japan Primary Care Association. Through abductive coding, we examined how the term was employed and the different meanings attributed to it.

Results: The instances of using the term "social" were classified into five distinct categories: (i) non-medical, (ii) emphasizing the importance of topics, (iii) public as an object, (iv) connections with people who support health and well-being, and (v) structural inequities that are detrimental to health.

Conclusion: The analysis revealed that the term "social" in the context of primary care discourse was multifaceted and characterized by ambiguity. To ensure effective communication and clarity in discussions, it is crucial for primary care professionals to have a clear understanding of the intended meaning and implications of the term "social."

## Introduction

Primary care professionals attend to patients’ health needs in both proximal and distal contexts [1], and are responsible not only for individual patients but also for the health of the community [2], necessitating their role as advocates [3]. Therefore, the importance of incorporating a "social” perspective in primary care practice has been emphasized [4]. 

This perspective is also emphasized in Japan. The concept of "social" is highly emphasized in the rubric of the Japan Primary Care Association's certified family physician residency program [5]. This is demonstrated by the incorporation of the term in various areas, such as "comprehensive information collection, integrated evaluation, and decision making in complex and difficult cases, not only in biomedical but also physiological and social aspects," "appropriate treatment and management based on psychosocial context, and assessment of symptoms and life changes," and "the improvement of the quality of life of patients and their families facing illness-related problems by early identification, assessment, and treatment of physical, psychosocial, and spiritual pain and problems to prevent or alleviate suffering."

Although the term "social" is commonly used in academic literature, researchers seldom explicate their specific comprehension of the term "social". To the best of our knowledge, only one study examining the utilization of "social" in medical research has shown that the term "social" is used in a highly polysemous manner in mental health research [6]. 

After the opening of Japan to the West at the end of the Edo period (1603-1868), various concepts from the West, including "society," were introduced to Japan. Society was not equivalent to the small-scale patriarchal order of Confucianism that had existed in Japan. Instead, it referred to an order of varied associations with public activities across a wide range of domains. As the concept of society gained acceptance, it was commonly translated as "shakai" (社会), a Chinese word that refers to a group (kai: 会) centered around a local guardian deity (sha: 社) [7]. The term that corresponds to "social" in Japanese is "shakai-teki" (社会的). "Teki" (的) is a suffix employed to form adjectives in Japanese. Hence, the word "shakai-teki" is now used in Japan as a one-to-one correspondence with "social." Currently, the terms "shakai" and "shakai-teki" have become so ubiquitous that Japanese speakers are unaware that they were initially used as translations [7].

Social themes tend to be perceived ambiguously and undervalued in importance in medical education [8]. Given that primary care emphasizes practice and knowledge in social perspectives, it is crucial to clearly understand what "social" denotes in the primary care discourse space. If the term is used ambiguously, this could obscure what primary care ought to prioritize. This study aims to elucidate how "social" is utilized in primary care discourse space in Japan.

## Materials and methods

Materials

The Japan Primary Care Association (JPCA), founded in 2010, is the leading academic organization for primary care in Japan. JPCA has administered a family physician residency program [9], and the program is accredited by the World Organization of Family Doctors. JPCA publishes three journals, including the Official Journal of the Japan Primary Care Association, which features scholarly articles and discussions on primary care in Japanese. This journal has been published quarterly since May 2010 and has produced 810 articles as of 2022. As a representative forum for primary care research and practice in Japan, this journal was considered appropriate for use as the material for this study.

Reflectivity

The first author is a primary care physician and Ph.D. student majoring in medical education. The second, third, and fourth authors are experts in medical education.

Data collection

Out of the total of 810 papers, the first author identified and selected papers that included the term "social" throughout their content. All selected papers were read in their entirety, and all usage of "social" were identified. The following uses were excluded: (i) quotes from foreign literature, (ii) reference lists, and (iii) terms appearing within specific institutions, laws, or other proper nouns. Additionally, terms used as repetitions in the same literature (e.g., the term "social problem" used repeatedly with the same meaning within a single paper) were grouped together as one. 

Data analysis

To investigate the meaning and application of each instance of the term "social," we utilized abductive coding [10]. This qualitative data analysis method involves constructing new theories based on unexpected discoveries that challenge existing theories. The researchers familiarized themselves with existing theories, and then concentrated on noteworthy discoveries, utilizing imaginative thinking to develop new theories to explain them. These theories were then verified by revisiting the data [10].

The process of abductive coding involved a combination of inductive coding and deductive coding. First, the first author conducted inductive coding for all instances of the term, taking into account the context in which it appeared. It was determined that each usage should be categorized based on the intended meaning. Next, deductive coding was employed, based on previous findings [6], to consider any instances that were not sufficiently addressed in the initial coding. The codes were then reviewed and revised to ensure accuracy in accordance with the data. Finally, the second, third, and fourth authors reviewed and discussed the coding process, iterating back and forth between data and theory until completion. We use Microsoft Excel 2021 for analysis.

Ethical considerations

This study was approved by the Institutional Review Board of the University of Tokyo (2022031NIe).

## Results

Out of the total of 810 papers, 184 papers included the term "social." After applying the exclusion criteria, 14 papers were ruled out, leaving a final analysis of 170 papers with a total of 243 instances of the term "social." These instances were analyzed, coded, and classified into five distinct categories as shown in Table 1.

**Table 1:** The categories about the usage of the term “social”

Category	The number of instances (percentage)
Non-medical	101 (41.6%)
Single use	40
Psychosocial	38
Bio-psycho-social	17
Physical, psychological, social, and spiritual	6
Emphasizing the importance of topics	45 (18.5%)
The importance of primary care	25
The importance of other issues	20
Public as an object	35 (14.4%)
Connections with people who support health and well-being	32 (13.2%)
Structural inequities that are detrimental to health	30 (12.3%)

All quotations below were originally written in Japanese and translated into English by the authors. Square brackets in the quotes denote researchers’ supplements. The list of quote sources is shown in Table 2.

**Table 2:** The list of quote sources

Quote sources (These quotes are translated from the Japanese language)
1. Sakanishi Y, Ouchi K, Tamai M, Nagata H, Ishibashi S, Sugioka S. Activity report of the medical shelter, short stay base based on regional alliance in Ishinomaki area after the Great East Japan Earthquake. Off J Japan Prim Care Assoc. 2015;38:108-112. [11]
2. Gohara S. Factors that influence the expression of opinions regarding choices in end-of-life medical services by community-dwelling elderly people. Off J Japan Prim Care Assoc. 2021;44(2):38-44. [12]
3. Kanada A, Kanoya Y. End-of-life care management for elderly persons requiring long-term care at home by medical and care professionals. Off J Japan Prim Care Assoc. 2021;44(2):74-80. [13]
4. Yokobayashi K, Matsuda S, Ushio K, et al. Woman in her twenties complaining of dizziness and head congestion: clinical jazz. [Memai/zuomokan wo shuso ni jushin shita 20 saidai josei: kurinikaru jazu.] Off J Japan Prim Care Assoc. 2012;35(1):76-82. [14]
5. Nakazawa Y, Stanyon M, Kanke S, Ii M, Kassai R. Reflections on Canadian family doctor training: Observation of postgraduate family medicine education with a focus on digital review for trainee development. Off J Japan Prim Care Assoc. 2021;44(1):20-22. [15]
6. Yamamoto R. Evaluation and treatment of cancer pain. [Gan toutuu no hyouka to chiryou.] Off J Japan Prim Care Assoc. 2014;37(2):178-181. [16]
7. Tokuda Y. Editorial: Special feature of the first issue. [Soukangou tokusyuu ni atatte.] Off J Japan Prim Care Assoc. 2010;33(2):123. [17]
8. Takada D, Matsuda H. A study showing changes in autonomic nervous system activity and relaxation in the elderly induced by “pleasant conversation” compared to “reading aloud.” Off J Japan Prim Care Assoc. 2013;36(1):5-10. [18]
9. Ikeda N, Yoshida T, Yamada S, Ishikawa M, Kamiya T. Different outcomes in two cases of idiopathic normal pressure hydrocephalus in the elderly living alone diagnosed by internists during hospitalization. Off J Japan Prim Care Assoc. 2019;42(1):52-57. [19]
10. Gohara S, Susa K. Qualitative analysis of opinions on the decisions made by community-dwelling elderly people regarding their medical treatment and care in the end-of-life. Off J Japan Prim Care Assoc. 2022;45(4):108-115. [20]
11. Haruta J. Recursion of "co-construction discourse" that is necessary for an inclusive community. Off J Japan Prim Care Assoc. 2020;43(1):1. [21]
12. Morimura K, Sugimoto T. General internal medicine conference in National Hospital Organization Higashi-Ohmi General Medical Center: 86-year-old woman complaining anorexia and thirst lasting for two weeks. [Kokuritsu byion kikou higashi oumi sougou iryou sentaa sougounaika kanfarensu: 2 syuukan mae kara tuzuku shokushi hushin koukatu wo uttaeru 86 sai josei.] Off J Japan Prim Care Assoc. 2013;36(3):249-258. [22]
13. Miyamoto K. Study on the occurrence of accrued receivables related to medical expenses: Initial analysis using accrued receivable data of Matsue Seikyo General Hospital. Off J Japan Prim Care Assoc. 2018;41(4):163-168. [23]
14. Mizumoto J, Komatsu M, Nagamine Y, Haseda M, Fujiwara K. How to prepare a showcase portfolio on social determinants of health. Off J Japan Prim Care Assoc. 2022;45(1):36-39. [24]

Non-medical (101 instances: 41.6%)

This category encompasses instances where the term "social" simply refers to non-medical factors or issues, often in a vague manner. Within this category, there were 40 instances where the term "social" was used independently.

Following the Great East Japan Earthquake, a medical evacuation center known as the Short Stay Base was established in the Ishinomaki area during the chronic phase of the disaster. The Primary Care for All Team (PCAT) assumed responsibility for its operation, serving as an institution catering to the needs of victims requiring not only medical care but also social support. (Quote 1)

Older people in the final stages of life repeatedly face important choices and decisions regarding various issues, such as selecting their end-of-life residence and care. These choices and decisions cannot be adequately addressed through medical knowledge alone but necessitate ethical and social perspectives. (Quote 2)

The term "social" was occasionally utilized to signify aspects related to daily living.

In addition to conducting general assessments, I propose a care plan that considers patients' living conditions in the future and considers their social situations. (Quote 3)

The term "psychosocial" (shinri-shakai-teki) was also employed in this context (38 instances).

It is crucial to ascertain the psychosocial background and the interpretive framework of the patient, rather than simply addressing it [=functional dizziness] superficially, with statements like "Nothing wrong, it’s all in your mind." (Quote 4)

Furthermore, the term was utilized in conjunction with the cliché "bio-psycho-social" or juxtaposed with "biomedical" and "social" (17 instances).

The resident was devoted to actively listening to patients and gathering bio-psycho-social information. (Quote 5)

Additionally, it was compared to "physical," "psychological," and "spiritual" (six instances.) 

Alleviating pain is a crucial subject in cancer care because it impacts not only physical suffering but also psychological, social, and spiritual well-being. (Quote 6)

Emphasizing the importance of topics（45 uses: 18.5%）

The term "social" was utilized to underscore the importance of specific topics and their influence on various stakeholders. Within the category, 25 instances highlighted the importance of primary care, advocating for its beneficence.

I believe that we have a vital social mission to strongly emphasize this matter [the importance of establishing a healthcare system with generalists at its core] to all sectors. (Quote 7)

There were 20 instances in the context of asserting the importance of addressing the issue.

Under such circumstances [=the circumstance of an increasing number of older individuals with dementia], the preservation of cognitive functions is crucial, and the effect of reading aloud [the subject of this research] holds immense social significance. (Quote 8)

Public as an object (35 instances: 14.4%)

The term "social" was used in a value-neutral manner, referring to the general public without specific reference to the primary care context.

Japan has the highest percentage of individuals aged 65 years or older worldwide, with potentially over 1 million patients with idiopathic normal pressure hydrocephalus (iNPH). Given this social background, general internists and emergency physicians are anticipated to encounter an increasing number of iNPH patients during their initial clinical encounters with older patients residing alone. (Quote 9)

However, 55.1% of Japanese citizens reported never discussing medical treatment and care during their end-of-life stage, indicating that the concept [of advance care planning] lacks widespread social awareness. (Quote 10)

Connections with people who support health and well-being（32 instances: 13.2%）

This category emphasized the positive implications of the term "social," highlighting the presence of interpersonal networks that contribute to the overall health and well-being of individuals.

When we carefully observe and attentively listen to others, we are enriched with expressions that foster connections among us. Primary care research sheds light on the reality that time, people, and social affluence cultivates such experiences in an inclusive community. (Quote 11)

The cooperation of patients [with diabetes] in their treatment, adequate social support, and the presence of comorbidities may also influence the treatment goals. (Quote 12)

Structural inequities that are detrimental to health (30 instances: 12.3%)

Instances in this category are related to the structural factors that adversely affect the health and well-being of individuals. Examples included terms such as "social isolation" (five instances,) "social vulnerability" (five instances,) and "social determinants of health" (five instances.)

The challenge lies in comprehending the social context of patients who face financial obstacles in affording medical examinations. (Quote 13)

Addressing patients' unmet social needs is an expected role of family physicians. (Quote 14)

## Discussion

This study revealed the multifaceted usage and significance of the term "social" within the primary care discourse. Few papers contained a clear explanation, which suggested that the term was used ambiguously. The usage of "social" in the sense of non-medical seems to be influenced by Engel's biopsychosocial model, as evidenced by the frequent use of the terms "psychosocial" or "bio-psycho-social" [25]. This model is sometimes interpreted reductionistically [26]. This usage seems to emerge from the temptation to refer to non-medical patient information collectively as "social." In addition, the term "social" was sometimes compared to "physical, " "psychological," and "spiritual," which seem to be influenced by the concept of total pain in palliative care [27,28].

An alternative application of the term "social" implies that the health and well-being of individuals are influenced by their surroundings, communities, and cultures. This usage is also evident in terms such as "social support," "social care," "social prescribing," and "social epidemiology." When the term is employed in a positive context to denote factors that contribute to an individual's health, it conveys a sense of interpersonal support. Conversely, when used to describe factors that impede health, it implies the presence of structural obstacles. The term "social" is also employed as an accentuation of the importance of a particular topic, in a distinct manner from the aforementioned usages. Specifically, the usage that highlights the role and importance of primary care professionals merits special attention. Primary care physicians often face disproportionate evaluation and scrutiny regarding their skills and responsibilities [29]. In Japan, unwarranted criticism from specialists can frequently undermine the professional identity of primary care professionals [30]. This usage may reflect the ambiguous professional identity of primary care professionals in Japan.

This study has some limitations. First, the instances gathered were solely from a single journal. We conducted a review of additional journals pertaining to Japanese primary care, including gray literature, and discovered that they contained numerous articles authored by non-primary care professionals. Second, this study exclusively encompassed literature written in Japanese, and all the researchers were native Japanese speakers. Consequently, articles written in languages other than Japanese were not examined, and the findings may be difficult to extrapolate to other linguistic contexts. Third, our focus was solely on the term "social" and did not encompass an analysis of the usage of the term "society". The term "society" may entail a broader significance. Furthermore, co-occurring expressions were not analyzed. Further research is warranted to gather a more extensive range of instances and scrutinize their usage in alternative languages. Fourth, the authors conducted the initial analysis of the data in Japanese and subsequently translated the codes and quotations into English. Despite the authors' considerable experience in translating medical literature, it is acknowledged that certain nuances may undergo alteration during the translation process.

## Conclusions

The utilization of the term "social" in the context of primary care discourse in Japan was characterized by its multifaceted and ambiguous nature. At times, it denoted non-medical factors, while in other instances, it conveyed the idea that individuals' health and well-being are impacted by their environment, both positively and negatively. Furthermore, the term was employed to emphasize the significance of specific topics, particularly highlighting the role of primary care. To ensure clarity and precision in their discussions and assertions, it is imperative for primary care professionals to have a clear understanding of the intended meaning of the term "social."
